# Size effects on the mechanical behavior and the compressive failure strength of concrete: an extensive dataset

**DOI:** 10.1016/j.dib.2020.106477

**Published:** 2020-11-02

**Authors:** Chi-Cong Vu, Jérôme Weiss, Olivier Plé, David Amitrano

**Affiliations:** a National University of Civil Engineering (NUCE), 55 Giai Phong, Hanoi, Vietnam; b Univ. Grenoble Alpes, CNRS, IRD, IFSTTAR, ISTerre, 38000 Grenoble, France; c Univ. Savoie Mont Blanc, CNRS, LOCIE, 73000 Chambéry, France

**Keywords:** Concrete, Compressive strength, Mechanical behavior, Size effects, Elastic modulus, Quasi-brittle materials

## Abstract

This data article provides a series of 492 stress-strain curves and compressive strength values obtained under the uniaxial compression of concrete samples fabricated from three different normal-weight concrete mixtures with four different cylindrical sample sizes ranging from 40 × 80 mm to 160 × 320 mm. These data are related to two research articles: “Revisiting statistical size effects on compressive failure of heterogeneous materials, with a special focus on concrete” (Vu et al., 2018) [1] and “Revisiting the concept of characteristic compressive strength of concrete” (Vu et al., 2020) [2]. In those papers, the strength values were used to (i) analyze and interpret statistical size effects on compressive strength of concrete (in ref. [1]), and (ii) discuss and evaluate the genuine characteristic compressive strength of concrete when size effects on strength are taken into account (in ref. [2]). This dataset could be reused for other statistical analyses on the mechanical behavior of concrete (e.g. elastic and strength properties) and associated possible mixture or size effects. In addition, the characteristic properties of the hardened concrete samples such as the apparent density, the moisture content, the modulus of elasticity as well as the internal microstructures are also provided.

## Specifications Table

**Table:** 

Subject	Engineering
Specific subject area	Mechanics of Materials, Civil engineering
Type of data	Tables Figures Text (.txt) files
How data were acquired	Uniaxial compression tests were carried out under a load-control protocol on 527 concrete samples by compression machines certified by the French Accreditation Committee (COFRAC). For 492 of these loading tests, the axial displacement during loading was measured by using one LVDT attached on the frame of the compression machine. The effect of the elastic deformation of the loading frame as a function of the applied load was corrected, using calibration tests on reference samples of known elastic moduli (aluminum and stainless steel). Image analysis of microstructures was performed from scanned images of internal sections of 12 hardened concrete samples (one sample for each concrete mixture and sample size). This scan procedure was done by using a flatbed scanner. An Acoustic Emission (AE) system manufactured by Physical Acoustics Corporation (PAC) was used to measure the P-wave velocity within a selection of 36 undamaged concrete specimens (three samples for each size and type of concrete), which was then used to determine the dynamic elastic properties (dynamic Young's modulus). The AE pulses were generated by breaking a pencil lead on one end of the concrete cylinder, and then captured by AE piezoelectric sensors of type PICO.
Data format	Raw Analyzed
Parameters for data collection	For large sample sizes (concrete cylinders of 70 mm,110 mm, and 160 mm in diameter), a compression machine with a loading capacity of 3000 kN was used, with a stiffness 2.9 times larger than that of the largest samples. The other machine, with a loading capacity of 300 kN, was used for the smallest samples (concrete cylinders of 40 mm in diameter). Its stiffness was 3.5 times larger than these small samples. A loading speed of 0.5 MPa/s was applied for all the tests and kept constant up to failure. During loading, force and displacement (hence, stress and strain) were recorded and monitored. All compression tests were performed after a minimum concrete's age of 28 days. To capture the images of concrete microstructure, a resolution of 1200dpi (corresponding to a 21.17μm pixel size) was used for scanning the internal sections of the concrete samples. For the Acoustic Emission measurements, a gain of 40 dB for the preamplifier and a detection threshold of 30 dB were applied in all the tests.
Description of data collection	A series of 492 stress-strain records and compressive failure strength values of concrete cylindrical samples fabricated from three different concrete mixtures and four different sample sizes were collected from uniaxial compression tests. An additional set of 35 failure strength values were collected for 160 mm-diameter samples of one type of concrete (i.e. C-concrete mixture): as the LVDT measurements were not correctly recorded for those tests due to a technical problem, the corresponding stress-strain records for these 35 samples are not provided. A pair of mean value and its standard deviations of the following parameters: apparent density, moisture content, P-wave velocity, dynamic elastic moduli, porosity, as well as the characteristic parameters of the internal microstructure for an individual concrete mixture and sample size is presented in this data article. The mean value of each parameter for a given sample size and type of concrete is calculated by averaging all the corresponding data of this parameter over its number of data. For a total, twelve (12) pairs of these two values above for a specified parameter of all concrete groups and all sample sizes
	are reported. The number of data is: 527 for the apparent density (about 43 values for each concrete mixture and sample size); 120 for the moisture content (10 values for each sample size and concrete mixture); 360 for the P-wave velocity and the dynamic elastic modulus (30 values for an individual concrete group and sample size); 72 for the porosity as well as the characteristic parameters of the internal microstructure of hardened concrete samples (6 values for each sample size and each concrete group).
Data source location	The dataset presented in this paper was collected from two locations: - Institut des Sciences de la Terre (ISTerre), 1381 rue de la Piscine, 38610 Gières, France; - IUT Chambéry, Univ. Savoie Mont Blanc (USMB), Savoie Technolac, 73376 Le Bourget du Lac Cedex, France.
Data accessibility	Repository name: Mendeley Data Data identification number: 10.17632/rnjnjvb3ph.1 Direct URL to data: https://data.mendeley.com/datasets/rnjnjvb3ph/draft?a=0df5c6e5-d3d2-4330-8274-14059b70e16e
Related research article	C.C. Vu, J. Weiss, O. Plé, D. Amitrano, D. Vandembroucq, Revisiting statistical size effects on compressive failure of heterogeneous materials, with a special focus on concrete, J. Mech. Phys. Solids. 121 (2018) 47–70. https://doi.org/10.1016/j.jmps.2018.07.022. C.C. Vu, O. Plé, J. Weiss, D. Amitrano, Revisiting the concept of characteristic compressive strength of concrete, Constr. Build. Mater. 263 (2020) 120126. https://doi.org/10.1016/j.conbuildmat.2020.120126.

## Value of the Data

• This dataset on compressive strength, of unprecedented size (527 mechanical tests) has a practical importance for the analysis of statistical size effects on compressive strength of concrete, a typical quasi-brittle material of tremendous importance in civil engineering.
• These data can serve as a reference strength dataset for normal-weight concrete, which could be used together with strength data for other types of concrete such as lightweight, high-strength, self-compacting concrete for an analysis of the influence of microstructures on strength, associated size effects, or mechanical behavior.
• These data can be used for analyzing the probability distribution of compressive strength, which is e.g. of upmost importance to determine the characteristic compressive strength of concrete.
• These data can be used for investigating the effect of concrete mix and sample size on the elastic properties of concrete.
• These data can be used together with other similar experimental campaigns performed under different testing conditions (temperature, humidity, etc.) to explore the influence of the moisture content on elastic properties, strength, and associated size effects.

## 1 Data Description

This data article presents the dataset obtained during the experimental campaign that was used in: [1] to analyze the statistical size effects on the compressive failure strength $\left(\sigma_{f}\right)$ of concrete in terms of mean strength, associated variability, and probability distribution; and to investigate the influence of microstructural disorder on the size effects of strength; and in [2] to discuss and evaluate the genuine characteristic compressive strength of concrete by taking into account the size effects on strength.

Three types of data are provided and given in the present paper. The first one contains the analyzed data of: (i) different characteristic properties of hardened concrete samples (i.e. apparent density, moisture content, modulus of elasticity); and (ii) the microstructural characteristics of our undamaged concrete samples. The data of point (i) are reported in Table 1, while those of point (ii) are reported in Table 2. In these tables, the values for a given sample size, for each concrete mixture, were averaged over all concrete samples of this examined size. (iii) The third dataset provides the dimensions, the mass, the density as well as the uniaxial compressive failure strength for 527 concrete samples (all sample sizes and concrete mixtures); see Table 3 to Table 14. Finally, among these 527 tests, we provide 492 stress-strain records. The corresponding data files (.txt) can be found in the Supplementary materials (SM) of this paper. For the remaining 35 compression tests of one type of concrete (i.e. 160 mm-diameter samples of C-concrete mixture), a technical problem did not allow to record the LVDT data, hence the strain records are not provided.

**Table 1:** Density, moisture content, P-wave velocity, dynamic elastic moduli of different sample sizes and different concrete mixtures.

		Density, ρ (kg/m^3^)		Moisture content, $w_{c}$ (%)		P-wave velocity, $V_{p}$ (m/s)		Dynamic elastic modulus, $Y_{d}$ (GPa)	

Concrete mixture	Sample size, ϕ×h (mm x mm)	Mean	SD	Mean	SD	Mean	SD	Mean	SD
F	40 × 80	2204.1	20.7	5.4	0.5	3798.0	164.5	29.4	2.6
	70 × 140	2201.1	11.0	5.4	0.4	3808.1	142.6	28.9	2.2
	110 × 220	2177.7	25.0	5.2	0.5	3823.7	121.7	28.6	2.1
	160 × 320	2153.1	25.3	5.0	0.2	3782.4	135.5	27.9	2.0
M	40 × 80	2415.3	16.5	3.4	0.3	4343.9	211.2	42.0	4.3
	70 × 140	2397.4	11.4	3.4	0.3	4337.0	134.4	40.9	2.6
	110 × 220	2386.3	18.6	3.0	0.1	4268.6	225.7	39.5	4.4
	160 × 320	2366.4	12.4	2.9	0.3	4283.0	187.9	39.1	3.5
C	40 × 80	2415.1	25.2	3.4	0.4	4168.7	293.9	38.1	5.3
	70 × 140	2421.6	11.7	3.4	0.4	4476.2	166.0	43.8	3.2
	110 × 220	2393.9	17.6	3.0	0.2	4359.4	262.4	41.0	5.0
	160 × 320	2384.6	18.0	3.1	0.2	4365.5	252.3	40.5	4.9

**Table 2:** The values of the microstructural characteristics length scales, $\xi_{g}$ and $\xi_{p}$, integral ranges $X_{0,g}$ and $X_{0,p}$, diameter of pore ($\langle d_{p}\rangle$ and $d_{p,\text{max}}$), and porosity averaged over all sections of each size of sample, for the 3 different concrete mixtures.

		$\xi_{g}$ (mm)		$X_{o,g}$ (mm)		$\xi_{p}$ (μm)		$X_{o,p}\;$(mm)		Mean pore diameter, 〈dp〉 (mm)		Maximum pore diameter, $d_{p,\max}$ (mm)		Porosity, $p_{o}$ (%)	

Concrete mixture	Sample size, ϕ×h (mm x mm)	Mean	SD	Mean	SD	Mean	SD	Mean	SD	Mean	SD	Mean	SD	Mean	SD
F	40 × 80	0.5	0.1	5.1	0.8	21.6	9.0	4.2	3.8	0.31	0.25	3.9	0.9	5.3	1.2
	70 × 140	0.6	0.1	5.7	0.6	27.4	3.4	5.2	4.1	0.34	0.30	3.7	0.5	6.2	0.5
	110 × 220	0.6	0.0	6.3	0.8	26.0	8.8	6.7	3.7	0.31	0.18	6.5	1.8	3.7	0.9
	160 × 320	0.6	0.0	11.1	5.8	30.6	6.0	11.3	5.7	0.37	0.25	6.9	1.2	3.9	0.7
M	40 × 80	2.0	0.4	9.7	2.5	9.0	7.5	2.7	0.7	0.28	0.21	3.1	0.5	1.6	0.2
	70 × 140	2.0	0.1	14.0	7.0	9.5	6.8	3.3	0.6	0.29	0.20	4.5	0.9	1.9	0.6
	110 × 220	2.3	0.3	15.1	3.3	13.3	3.3	6.1	2.1	0.34	0.22	6.7	1.2	1.9	0.5
	160 × 320	2.4	0.2	16.1	4.1	7.4	3.2	5.4	2.7	0.31	0.23	5.2	1.1	1.1	0.3
C	40 × 80	3.5	0.8	10.0	1.1	6.1	3.3	5.5	4.2	0.23	0.21	2.5	0.6	1.1	0.4
	70 × 140	3.5	0.3	15.9	3.9	8.6	4.4	4.5	1.7	0.27	0.18	4.1	1.0	1.7	0.6
	110 × 220	3.2	0.3	22.5	11.1	8.9	4.7	6.9	3.5	0.33	0.23	5.4	1.3	1.4	0.5
	160 × 320	3.8	0.5	27.8	6.7	10.5	4.9	7.1	3.4	0.29	0.19	5.3	1.0	1.6	0.3

**Table 3:** The details of dimensions, mass, density, and the compressive failure strength for different cylindrical (ϕ×h = 40 × 80 mm) concrete samples of F-concrete.

	Sample	Sample dimensions	Height,	Mass of	Density,	Peak load,	Compressive failure
No.	ID	Diameter, ϕ (mm)	h (mm)	sample (g)	ρ (kg/m^3^)	$F_{\max}$ (kN)	strength, $\sigma_{f}$ (MPa)
1	F4-B1-1	39.0	81.0	213.0	2201.3	66.5	55.7
2	F4-B1-2	39.0	81.0	216.0	2232.3	81.0	67.8
3	F4-B1-3	39.0	81.0	214.0	2211.6	67.9	56.8
4	F4-B1-4	39.0	81.0	216.0	2232.3	72.2	60.4
5	F4-B1-5	39.0	81.0	214.0	2211.6	66.5	55.6
6	F4-B1-6	39.0	81.0	216.0	2232.3	70.7	59.2
7	F4-B1-7	39.0	81.0	215.0	2222.0	76.0	63.7
8	F4-B1-8	39.0	81.0	214.0	2211.6	60.3	50.4
9	F4-B1-9	39.0	80.5	208.0	2163.0	68.6	57.4
10	F4-B1-10	39.0	80.5	207.0	2152.6	80.5	67.4
11	F4-B1-11	39.0	81.0	211.0	2180.6	85.0	71.2
12	F4-B1-12	39.0	81.0	212.0	2190.9	70.4	59.0
13	F4-B1-13	39.0	81.0	212.0	2190.9	65.6	54.9
14	F4-B1-14	39.0	80.5	214.0	2225.4	77.6	64.9
15	F4-B1-15	39.0	81.0	210.0	2170.3	70.2	58.8
16	F4-B1-16	39.0	81.0	214.0	2211.6	66.4	55.6
17	F4-B1-17	39.0	80.5	213.0	2215.0	61.9	51.8
18	F4-B1-18	39.0	81.0	214.0	2211.6	67.2	56.3
19	F4-B1-19	39.0	81.0	215.0	2222.0	85.2	71.3
20	F4-B1-20	39.0	81.0	214.0	2211.6	60.8	50.9
21	F4-B1-21	39.0	81.0	214.0	2211.6	71.8	60.1
22	F4-B1-22	39.0	80.5	213.0	2215.0	70.3	58.9
23	F4-B1-23	39.0	81.0	215.0	2222.0	70.5	59.0
24	F4-B1-24	39.0	81.0	214.0	2211.6	72.6	60.8
25	F4-B1-25	39.0	80.5	213.0	2215.0	71.0	59.5
26	F4-B1-26	39.0	81.0	214.0	2211.6	59.3	49.6
27	F4-B1-27	39.0	81.0	213.0	2201.3	74.7	62.5
28	F4-B1-28	39.0	81.0	214.0	2211.6	76.2	63.8
29	F4-B1-29	39.0	81.0	212.0	2190.9	76.3	63.9
30	F4-B1-30	39.0	81.0	213.0	2201.3	75.1	62.9
31	F4-B1-31	39.0	81.0	215.0	2222.0	80.9	67.8
32	F4-B1-32	39.0	81.0	215.0	2222.0	71.7	60.0
33	F4-B1-33	39.0	80.5	211.0	2194.2	69.0	57.8
34	F4-B1-34	39.0	80.5	205.0	2131.8	72.3	60.5
35	F4-B1-35	39.0	80.5	209.0	2173.4	69.5	58.2
36	F4-B1-36	39.0	81.0	213.0	2201.3	61.3	51.3
37	F4-B1-37	39.0	81.0	213.0	2201.3	60.8	50.9
38	F4-B1-38	39.0	81.0	214.0	2211.6	56.7	47.4
39	F4-B1-39	39.0	81.0	215.0	2222.0	55.4	46.4
40	F4-B1-40	39.0	81.5	215.0	2208.3	54.0	45.2
41	F4-B1-41	39.0	81.0	213.0	2201.3	53.3	44.6
42	F4-B1-42	39.0	81.0	213.0	2201.3	53.0	44.4
43	F4-B1-43	39.0	81.0	213.0	2201.3	52.2	43.7
44	F4-B1-44	39.0	81.0	211.0	2180.6	51.0	42.7
45	F4-B1-45	39.0	81.0	214.0	2211.6	50.4	42.2
46	F4-B1-46	39.0	81.0	214.0	2211.6	50.6	42.3

The values of the elastic properties and several characteristic parameters of hardened concrete samples, as well as the values of the compressive strength failure strength for all concrete samples are presented in 14 (fourteen) tables as follows:

• Table 1 represents the values of density, moisture content, P-wave velocity, dynamic elastic modulus of different sample sizes and different concrete mixtures. While the values of density and moisture content have been presented in Fig. 3 of ref. [2], those of P-wave velocity and dynamic elastic modulus have been displayed in Fig. 11 of ref. [1].
• Table 2 represents the values of the microstructural characteristics length scales, $\xi_{g}$ and $\xi_{p}$, integral ranges $X_{0,g}$ and $X_{0,p}$, diameter of pore, and porosity averaged over all sections of each size of sample, for the three concrete mixtures. While the values of $\xi_{g}$ and $\xi_{p}$ have been shown in Fig. 6 and Fig. 9b respectively of ref. [1], the diameter of pore and porosity for the three different concrete mixtures have been presented in Table A1 of ref. [2].
• Table 3 represents the details of dimensions, mass, density, and the compressive failure strength for different cylindrical (ϕ×h=40×80mm) concrete samples of F-concrete.
• Table 4 represents the details of dimensions, mass, density, and the compressive failure strength for different cylindrical (ϕ×h=70×140mm) concrete samples of F-concrete.

**Table 4:** The details of dimensions, mass, density, and the compressive failure strength for different cylindrical (ϕ×h = 70 × 140 mm) concrete samples of F-concrete.

		Sample dimensions					

No.	Sample ID	Diameter, ϕ (mm)	Height, h (mm)	Mass of sample (g)	Density, ρ (kg/m^3^)	Peak load, $F_{\max}$ (kN)	Compressive failure strength, $\sigma_{f}$ (MPa)
1	F7-B1-1	69.5	139.0	1156.0	2192.2	184.9	48.7
2	F7-B1-2	69.5	140.0	1173.0	2208.6	185.6	48.9
3	F7-B1-3	69.5	139.5	1166.0	2203.3	183.7	48.4
4	F7-B1-4	69.5	140.0	1165.0	2193.5	214.3	56.5
5	F7-B1-5	69.5	140.0	1170.0	2202.9	200.9	52.9
6	F7-B1-6	69.5	140.0	1174.0	2210.4	154.0	40.6
7	F7-B1-7	69.5	139.0	1165.0	2209.3	171.1	45.1
8	F7-B1-8	69.5	140.0	1171.0	2204.8	160.9	42.4
9	F7-B1-9	69.5	140.0	1173.0	2208.6	175.1	46.2
10	F7-B1-10	69.5	139.5	1168.0	2207.0	167.1	44.1
11	F7-B1-11	69.5	140.0	1181.0	2223.6	216.6	57.1
12	F7-B1-12	69.5	139.5	1169.0	2208.9	167.2	44.1
13	F7-B1-13	69.5	140.0	1173.0	2208.6	220.3	58.1
14	F7-B1-14	69.5	140.0	1170.0	2202.9	209.8	55.3
15	F7-B1-15	69.5	139.0	1171.0	2220.7	211.9	55.9
16	F7-B1-16	69.5	140.0	1175.0	2212.3	172.1	45.4
17	F7-B1-17	69.5	139.0	1164.0	2207.4	191.4	50.4
18	F7-B1-18	69.5	140.0	1172.0	2206.7	163.9	43.2
19	F7-B1-19	69.5	140.0	1175.0	2212.3	221.7	58.4
20	F7-B1-20	69.5	139.5	1177.0	2224.0	187.9	49.5
21	F7-B1-21	69.5	140.0	1169.0	2201.0	216.8	57.1
22	F7-B1-22	69.5	139.0	1160.0	2199.8	189.2	49.9
23	F7-B1-23	69.5	139.5	1164.0	2199.5	218.8	57.7
24	F7-B1-24	69.5	140.0	1167.0	2197.3	152.2	40.1
25	F7-B1-25	69.5	140.0	1171.0	2204.8	170.0	44.8
26	F7-B1-26	69.5	140.0	1160.0	2184.1	174.0	45.9
27	F7-B1-27	69.5	140.0	1159.0	2182.2	209.0	55.1
28	F7-B1-28	69.5	140.0	1175.0	2212.3	202.3	53.3
29	F7-B1-29	69.5	140.0	1163.0	2189.7	162.7	42.9
30	F7-B1-30	69.5	140.0	1171.0	2204.8	172.4	45.4
31	F7-B1-31	69.5	139.5	1155.0	2182.5	175.0	46.1
32	F7-B1-32	69.5	140.0	1160.0	2184.1	171.2	45.1
33	F7-B1-33	69.5	139.0	1165.0	2209.3	208.1	54.8
34	F7-B1-34	69.5	140.0	1163.0	2189.7	197.7	52.1
35	F7-B1-35	69.5	140.0	1166.0	2195.4	166.8	44.0
36	F7-B1-36	69.5	139.5	1155.0	2182.5	193.5	51.0
37	F7-B1-37	69.5	139.5	1161.0	2193.8	168.4	44.4
38	F7-B1-38	69.5	140.0	1160.0	2184.1	193.5	51.0
39	F7-B1-39	69.5	140.0	1175.0	2212.3	204.4	53.9
40	F7-B1-40	69.5	140.0	1169.0	2201.0	182.2	48.0
41	F7-B1-41	69.5	140.0	1173.0	2208.6	211.6	55.8
42	F7-B1-42	69.5	141.0	1169.0	2185.4	176.8	46.6
43	F7-B1-43	69.5	140.0	1167.0	2197.3	199.5	52.6
44	F7-B1-44	69.5	140.0	1168.0	2199.2	204.8	54.0
45	F7-B1-45	69.5	140.0	1167.0	2197.3	181.0	47.7
46	F7-B1-46	69.5	139.5	1162.0	2195.7	211.3	55.7
• Table 5 represents the details of dimensions, mass, density, and the compressive failure strength for different cylindrical (ϕ×h=110×220mm) concrete samples of F-concrete.

**Table 5:** The details of dimensions, mass, density, and the compressive failure strength for different cylindrical (ϕ×h = 110 × 220 mm) concrete samples of F-concrete.

		Sample dimensions					

No.	Sample ID	Diameter, ϕ (mm)	Height, h (mm)	Mass of sample (kg)	Density, ρ (kg/m^3^)	Peak load, $F_{\max}$ (kN)	Compressive failure strength, $\sigma_{f}$ (MPa)
1	F11-B1-1	111.7	215.2	4.6	2193.6	423.3	43.2
2	F11-B1-2	111.8	216.0	4.6	2184.0	422.7	43.1
3	F11-B2-3	111.9	211.2	4.5	2184.8	429.3	43.7
4	F11-B2-4	112.0	211.5	4.6	2191.8	474.9	48.2
5	F11-B2-5	112.1	211.2	4.5	2167.0	432.9	43.9
6	F11-B2-6	111.9	213.2	4.6	2176.7	420.5	42.8
7	F11-B2-7	111.5	216.0	4.6	2173.5	422.6	43.3
8	F11-B2-8	111.9	219.0	4.6	2120.5	473.0	48.1
9	F11-B2-9	112.1	211.0	4.5	2180.1	450.5	45.6
10	F11-B2-10	111.9	213.8	4.6	2187.8	432.4	44.0
11	F11-B2-11	111.8	212.0	4.5	2178.1	440.3	44.8
12	F11-B2-12	112.2	207.3	4.5	2195.0	491.0	49.7
13	F11-B2-13	111.8	215.0	4.6	2189.9	421.7	43.0
14	F11-B2-14	112.0	215.0	4.6	2176.9	439.8	44.6
15	F11-B2-15	112.0	211.0	4.6	2196.0	477.0	48.4
16	F11-B2-16	111.8	213.0	4.6	2212.8	469.1	47.8
17	F11-B2-17	111.8	216.5	4.7	2188.3	390.0	39.7
18	F11-B2-18	112.0	214.0	4.6	2168.1	387.5	39.3
19	F11-B3-19	111.7	218.7	4.7	2172.1	444.7	45.4
20	F11-B3-20	112.0	216.0	4.6	2175.7	417.3	42.4
21	F11-B3-21	111.6	216.7	4.7	2213.5	457.4	46.8
22	F11-B3-22	111.8	219.0	4.6	2161.0	434.1	44.2
23	F11-B3-23	111.8	215.0	4.6	2165.7	453.5	46.2
24	F11-B3-24	111.8	213.7	4.6	2184.6	462.6	47.1
25	F11-B3-25	112.0	212.0	4.6	2183.2	450.1	45.7
26	F11-B3-26	111.8	217.3	4.6	2170.0	424.7	43.3
27	F11-B3-27	111.8	215.0	4.6	2195.1	436.3	44.4
28	F11-B3-28	111.6	217.8	4.6	2174.2	451.4	46.1
29	F11-B3-29	112.0	213.0	4.6	2183.5	441.5	44.8
30	F11-B3-30	112.0	216.0	4.6	2157.4	438.6	44.5
31	F11-B3-31	111.8	218.0	4.6	2135.0	447.0	45.5
32	F11-B3-32	111.8	211.5	4.6	2218.4	468.7	47.7
33	F11-B3-33	111.8	215.0	4.6	2192.2	421.1	42.9
34	F11-B3-34	111.8	217.0	4.8	2238.7	453.3	46.2
35	F11-B4-35	111.8	214.7	4.6	2179.2	421.0	42.9
36	F11-B4-36	112.0	215.0	4.5	2136.3	429.1	43.6
37	F11-B4-37	111.8	214.7	4.6	2180.6	429.9	43.8
38	F11-B4-38	112.2	217.8	4.6	2156.1	423.6	42.8
39	F11-B4-39	111.7	217.2	4.7	2186.1	406.5	41.5
40	F11-B4-40	111.8	210.8	4.6	2201.6	432.5	44.1
41	F11-B4-41	111.8	221.8	4.5	2084.2	415.7	42.3
42	F11-B4-42	111.8	213.3	4.5	2166.7	421.2	42.9
43	F11-B4-43	111.5	215.8	4.6	2176.4	376.7	38.6
44	F11-B4-44	111.6	217.0	4.6	2171.8	387.8	39.6
45	F11-B4-45	111.8	212.0	4.5	2157.4	434.2	44.2
• Table 6 represents the details of dimensions, mass, density, and the compressive failure strength for different cylindrical (ϕ×h=160×320mm) concrete samples of F-concrete.

**Table 6:** The details of dimensions, mass, density, and the compressive failure strength for different cylindrical (ϕ×h = 160 × 320 mm) concrete samples of F-concrete.

		Sample dimensions					

No.	Sample ID	Diameter, ϕ (mm)	Height, h (mm)	Mass of sample (kg)	Density, ρ (kg/m^3^)	Peak load, $F_{\max}$ (kN)	Compressive failure strength, $\sigma_{f}$ (MPa)
1	F16-B1-1	160.0	310.0	13.5	2172.8	851.2	42.3
2	F16-B1-2	160.0	315.0	13.5	2135.0	818.5	40.7
3	F16-B1-3	160.0	312.5	13.7	2178.5	855.7	42.6
4	F16-B1-4	160.0	312.0	13.5	2151.9	797.5	39.7
5	F16-B1-5	160.0	316.0	13.6	2137.2	784.5	39.0
6	F16-B1-6	160.0	315.0	13.5	2130.3	784.3	39.0
7	F16-B1-7	160.0	315.7	13.6	2135.5	835.5	41.6
8	F16-B1-8	160.0	311.7	13.5	2161.8	834.6	41.5
9	F16-B1-9	160.0	313.3	13.5	2138.0	777.1	38.6
10	F16-B2-10	160.0	314.0	13.5	2134.2	838.4	41.7
11	F16-B2-11	160.0	311.8	13.6	2162.8	862.6	42.9
12	F16-B2-12	160.0	315.0	13.4	2119.2	931.4	46.3
13	F16-B2-13	160.0	309.0	13.5	2180.7	906.4	45.1
14	F16-B2-14	160.0	311.3	13.4	2133.2	832.0	41.4
15	F16-B2-15	160.0	317.0	13.7	2154.2	834.1	41.5
16	F16-B2-16	160.0	309.0	13.4	2156.8	868.0	43.2
17	F16-B2-17	160.0	311.2	13.5	2160.9	883.4	43.9
18	F16-B2-18	160.0	308.7	13.4	2163.8	895.5	44.5
19	F16-B2-19	160.0	303.5	13.1	2154.3	897.5	44.6
20	F16-B2-20	160.0	306.7	13.5	2192.0	932.1	46.4
21	F16-B2-21	160.0	316.0	13.5	2118.0	788.5	39.2
22	F16-B3-22	160.0	310.0	13.3	2126.5	844.6	42.0
23	F16-B3-23	160.0	312.2	13.3	2124.5	817.0	40.6
24	F16-B3-24	160.0	310.0	13.5	2160.8	859.7	42.8
25	F16-B3-25	160.0	312.0	13.3	2124.1	830.3	41.3
26	F16-B3-26	160.0	314.8	13.4	2120.6	813.2	40.4
27	F16-B3-27	160.0	302.0	13.3	2188.9	920.6	45.8
28	F16-B3-28	160.0	311.3	13.3	2129.6	821.6	40.9
29	F16-B3-29	160.0	310.0	13.6	2177.8	909.8	45.3
30	F16-B3-30	160.0	316.0	13.5	2119.4	778.6	38.7
31	F16-B3-31	160.0	314.0	13.5	2132.3	768.4	38.2
32	F16-B3-32	160.0	307.0	13.5	2179.8	901.6	44.8
33	F16-B3-33	160.0	309.7	13.2	2124.2	828.3	41.2
34	F16-B3-34	160.0	303.2	13.4	2200.4	875.4	43.5
35	F16-B4-35	160.0	315.0	13.6	2141.8	792.4	39.4
36	F16-B4-36	160.0	310.0	13.5	2162.7	893.6	44.4
37	F16-B4-37	160.0	313.7	13.4	2120.7	793.3	39.5
38	F16-B4-38	160.0	315.8	13.5	2120.3	815.2	40.5
39	F16-B4-39	160.0	312.0	13.3	2123.7	816.0	40.6
40	F16-B4-40	160.0	315.0	13.9	2195.2	880.3	43.8
41	F16-B4-41	160.0	310.8	13.5	2166.6	821.9	40.9
42	F16-B4-42	160.0	308.0	13.4	2169.0	855.7	42.6
43	F16-B4-43	160.0	312.0	13.6	2174.8	891.0	44.3
44	F16-B4-44	160.0	306.5	13.6	2206.7	777.1	38.6
45	F16-B4-45	160.0	309.0	13.5	2171.0	780.1	38.8
46	F16-B4-46	160.0	310.0	13.6	2178.9	769.7	38.3
47	F16-B4-47	160.0	309.0	13.5	2180.2	944.6	47.0
• Table 7 represents the details of dimensions, mass, density, and the compressive failure strength for different cylindrical (ϕ×h=40×80mm) concrete samples of M-concrete.

**Table 7:** The details of dimensions, mass, density, and the compressive failure strength for different cylindrical (ϕ×h = 40 × 80 mm) concrete samples of M-concrete.

		Sample dimensions					

No.	Sample ID	Diameter, ϕ (mm)	Height, h (mm)	Mass of sample (g)	Density, ρ (kg/m^3^)	Peak load, $F_{\max}$ (kN)	Compressive failure strength, $\sigma_{f}$ (MPa)
1	M4-B1-1	39.0	81.0	234.0	2418.3	54.8	45.9
2	M4-B1-2	39.0	81.0	232.0	2397.6	58.4	48.9
3	M4-B1-3	39.0	81.0	235.0	2428.6	58.0	48.5
4	M4-B1-4	39.0	80.5	233.0	2422.9	67.0	56.1
5	M4-B1-5	39.0	80.5	231.0	2402.1	66.3	55.5
6	M4-B1-6	39.0	81.0	233.0	2408.0	56.8	47.6
7	M4-B1-7	39.0	81.0	235.0	2428.6	67.2	56.3
8	M4-B1-8	39.0	80.5	231.0	2402.1	59.9	50.2
9	M4-B1-9	39.0	81.0	233.0	2408.0	51.0	42.7
10	M4-B1-10	39.0	81.0	237.0	2449.3	68.5	57.3
11	M4-B1-11	39.0	80.0	231.0	2417.1	62.5	52.3
12	M4-B1-12	39.0	80.5	233.0	2422.9	62.4	52.3
13	M4-B1-13	39.0	80.0	232.0	2427.6	72.9	61.1
14	M4-B1-14	39.0	80.5	232.0	2412.5	60.7	50.8
15	M4-B1-15	39.0	80.0	231.0	2417.1	70.9	59.4
16	M4-B1-16	39.0	81.0	231.0	2387.3	63.5	53.2
17	M4-B1-17	39.0	81.5	236.0	2424.0	61.1	51.2
18	M4-B1-18	39.0	81.5	237.0	2434.3	54.9	46.0
19	M4-B1-19	39.0	82.0	236.0	2409.2	60.9	51.0
20	M4-B1-20	39.0	82.0	236.0	2409.2	50.4	42.2
21	M4-B1-21	39.0	82.0	237.0	2419.4	53.8	45.0
22	M4-B1-22	39.0	82.0	236.0	2409.2	60.5	50.7
23	M4-B1-23	39.0	82.0	235.0	2399.0	51.8	43.4
24	M4-B1-24	39.0	80.5	236.0	2454.1	73.0	61.1
25	M4-B1-25	39.0	80.5	232.0	2412.5	71.6	59.9
26	M4-B1-26	39.0	80.5	232.0	2412.5	68.6	57.4
27	M4-B1-27	39.0	80.5	233.0	2422.9	60.8	50.9
28	M4-B1-28	39.0	80.5	236.0	2454.1	67.3	56.4
29	M4-B1-29	39.0	81.0	233.0	2408.0	54.5	45.6
30	M4-B1-30	39.0	82.0	236.0	2409.2	63.2	52.9
31	M4-B1-31	39.0	82.0	236.0	2409.2	54.5	45.6
32	M4-B1-32	39.0	82.0	235.0	2399.0	59.8	50.1
33	M4-B1-33	39.0	80.5	228.0	2370.9	52.1	43.6
34	M4-B1-34	39.0	82.0	235.0	2399.0	50.2	42.0
35	M4-B1-35	39.0	81.5	236.0	2424.0	49.9	41.8
36	M4-B1-36	39.0	82.0	235.0	2399.0	51.5	43.1
37	M4-B1-37	39.0	82.0	237.0	2419.4	52.5	44.0
38	M4-B1-38	39.0	81.5	235.0	2413.7	69.3	58.0
39	M4-B1-39	39.0	81.0	235.0	2428.6	50.0	41.9
40	M4-B1-40	39.0	81.5	234.0	2403.5	50.1	41.9
41	M4-B1-41	39.0	80.5	233.0	2422.9	49.6	41.5
42	M4-B1-42	39.0	81.5	234.0	2403.5	49.4	41.4
43	M4-B1-43	39.0	81.5	238.0	2444.6	44.4	37.2
44	M4-B1-44	39.0	81.0	233.0	2408.0	45.6	38.2
• Table 8 represents the details of dimensions, mass, density, and the compressive failure strength for different cylindrical (ϕ×h=70×140mm) concrete samples of M-concrete.

**Table 8:** The details of dimensions, mass, density, and the compressive failure strength for different cylindrical (ϕ×h = 70 × 140 mm) concrete samples of M-concrete.

		Sample dimensions					

No.	Sample ID	Diameter, ϕ (mm)	Height, h (mm)	Mass of sample (g)	Density, ρ (kg/m^3^)	Peak load, $F_{\max}$ (kN)	Compressive failure strength, $\sigma_{f}$ (MPa)
1	M7-B1-1	69.5	140.0	1254.0	2361.1	189.0	49.8
2	M7-B1-2	69.5	140.0	1270.0	2391.2	189.6	50.0
3	M7-B1-3	69.5	140.0	1278.0	2406.3	163.9	43.2
4	M7-B1-4	69.5	140.0	1272.0	2395.0	161.0	42.4
5	M7-B1-5	69.5	140.0	1272.0	2395.0	180.3	47.5
6	M7-B1-6	69.5	140.0	1275.0	2400.6	170.6	45.0
7	M7-B1-7	69.5	140.0	1280.0	2410.0	179.4	47.3
8	M7-B1-8	69.5	140.0	1269.0	2389.3	165.5	43.6
9	M7-B1-9	69.5	140.0	1272.0	2395.0	198.1	52.2
10	M7-B1-10	69.5	140.0	1270.0	2391.2	170.6	45.0
11	M7-B1-11	69.5	139.5	1279.0	2416.8	187.4	49.4
12	M7-B1-12	69.5	140.0	1284.0	2417.6	185.4	48.9
13	M7-B1-13	69.5	140.0	1275.0	2400.6	187.5	49.4
14	M7-B1-14	69.5	140.0	1266.0	2383.7	189.1	49.8
15	M7-B1-15	69.5	140.0	1278.0	2406.3	138.7	36.5
16	M7-B1-16	69.5	139.0	1271.0	2410.3	142.6	37.6
17	M7-B1-17	69.5	139.5	1268.0	2396.0	142.2	37.5
18	M7-B1-18	69.5	140.0	1273.0	2396.9	141.1	37.2
19	M7-B1-19	69.5	142.0	1290.0	2394.6	191.9	50.6
20	M7-B1-20	69.5	142.0	1292.0	2398.4	182.4	48.1
21	M7-B1-21	69.5	142.0	1291.0	2396.5	199.5	52.6
22	M7-B1-22	69.5	134.0	1213.0	2386.1	181.8	47.9
23	M7-B1-23	69.5	142.0	1277.0	2370.5	179.3	47.3
24	M7-B1-24	69.5	142.0	1283.0	2381.7	187.2	49.3
25	M7-B1-25	69.5	142.0	1302.0	2416.9	189.8	50.0
26	M7-B1-26	69.5	142.0	1291.0	2396.5	175.8	46.3
27	M7-B1-27	69.5	142.0	1288.0	2390.9	177.6	46.8
28	M7-B1-28	69.5	142.0	1287.0	2389.1	175.8	46.3
29	M7-B1-29	69.5	142.0	1294.0	2402.1	171.8	45.3
30	M7-B1-30	69.5	142.0	1291.0	2396.5	169.2	44.6
31	M7-B1-31	69.5	142.0	1297.0	2407.6	169.8	44.7
32	M7-B1-32	69.5	142.0	1289.0	2392.8	180.4	47.6
33	M7-B1-33	69.5	142.0	1296.0	2405.8	161.4	42.6
34	M7-B1-34	69.5	131.0	1189.0	2392.5	180.2	47.5
35	M7-B1-35	69.5	132.0	1200.0	2396.3	174.1	45.9
36	M7-B1-36	69.5	142.0	1296.0	2405.8	166.2	43.8
37	M7-B1-37	69.5	142.0	1287.0	2389.1	181.5	47.9
38	M7-B1-38	69.5	142.0	1290.0	2394.6	170.1	44.8
39	M7-B1-39	69.5	142.0	1301.0	2415.1	153.6	40.5
40	M7-B1-40	69.5	142.0	1298.0	2409.5	157.3	41.5
41	M7-B1-41	69.5	142.0	1291.0	2396.5	146.9	38.7
42	M7-B1-42	69.5	142.0	1288.0	2390.9	142.6	37.6
43	M7-B1-43	69.5	142.0	1288.0	2390.9	141.1	37.2
44	M7-B1-44	69.5	140.0	1271.0	2393.1	138.1	36.4
45	M7-B1-45	69.5	139.0	1271.0	2410.3	152.4	40.2
• Table 9 represents the details of dimensions, mass, density, and the compressive failure strength for different cylindrical (ϕ×h=110×220mm) concrete samples of M-concrete.

**Table 9:** The details of dimensions, mass, density, and the compressive failure strength for different cylindrical (ϕ×h = 110 × 220 mm) concrete samples of M-concrete.

		Sample dimensions					

No.	Sample ID	Diameter, ϕ (mm)	Height, h (mm)	Mass of sample (kg)	Density, ρ (kg/m^3^)	Peak load, $F_{\max}$ (kN)	Compressive failure strength, $\sigma_{f}$ (MPa)
1	M11-B1-1	112.2	221.5	5.2	2379.4	421.0	42.6
2	M11-B1-2	111.8	221.5	5.2	2397.4	413.1	42.1
3	M11-B1-3	111.9	223.7	5.3	2392.8	443.2	45.1
4	M11-B1-4	112.6	218.7	5.1	2353.3	417.6	41.9
5	M11-B1-5	112.4	222.0	5.2	2371.1	419.9	42.3
6	M11-B1-6	111.4	223.5	5.3	2410.5	419.4	43.0
7	M11-B1-7	112.0	218.7	5.1	2384.6	407.5	41.4
8	M11-B1-8	112.3	219.7	5.1	2366.2	428.5	43.3
9	M11-B1-9	112.0	223.0	5.3	2395.5	439.9	44.7
10	M11-B2-10	112.3	219.5	5.1	2368.3	436.5	44.1
11	M11-B2-11	111.7	218.3	5.1	2373.8	401.2	40.9
12	M11-B2-12	111.8	221.0	5.2	2379.8	401.8	40.9
13	M11-B2-13	111.8	220.3	5.1	2379.5	417.1	42.5
14	M11-B2-14	112.1	218.8	5.1	2358.4	392.9	39.8
15	M11-B2-15	112.0	216.8	5.0	2362.0	389.3	39.5
16	M11-B2-16	111.9	221.7	5.1	2351.5	393.3	40.0
17	M11-B2-17	111.5	219.2	5.2	2411.3	445.0	45.6
18	M11-B2-18	111.8	218.0	5.1	2378.9	384.4	39.2
19	M11-B2-19	111.7	219.7	5.1	2387.5	408.6	41.7
20	M11-B2-20	111.4	220.0	5.2	2412.5	373.7	38.3
21	M11-B2-21	112.3	220.0	5.2	2364.3	416.6	42.1
22	M11-B3-22	111.8	218.7	5.1	2390.8	418.7	42.6
23	M11-B3-23	111.7	222.7	5.2	2396.1	424.2	43.3
24	M11-B3-24	111.7	218.0	5.1	2401.4	440.6	45.0
25	M11-B3-25	111.7	219.0	5.1	2379.3	431.2	44.0
26	M11-B3-26	111.5	221.5	5.2	2414.0	401.3	41.1
27	M11-B3-27	111.8	222.3	5.2	2403.9	428.7	43.7
28	M11-B3-28	111.9	223.0	5.2	2393.4	409.4	41.6
29	M11-B3-29	111.9	221.3	5.2	2390.7	425.3	43.3
30	M11-B3-30	111.6	221.0	5.2	2400.8	407.6	41.7
31	M11-B3-31	111.5	221.0	5.2	2407.9	392.2	40.2
32	M11-B3-32	112.2	221.8	5.2	2348.4	376.2	38.1
33	M11-B3-33	111.8	218.0	5.1	2376.1	421.6	42.9
34	M11-B4-34	111.8	219.0	5.2	2402.9	398.3	40.6
35	M11-B4-35	112.2	221.0	5.1	2350.0	441.4	44.6
36	M11-B4-36	112.0	219.7	5.2	2387.2	386.6	39.2
37	M11-B4-37	111.8	221.0	5.2	2379.3	424.2	43.2
38	M11-B4-38	112.0	223.0	5.3	2398.7	399.5	40.5
39	M11-B4-39	112.2	221.8	5.2	2381.2	401.6	40.6
40	M11-B4-40	112.1	220.7	5.2	2387.7	412.2	41.8
41	M11-B4-41	111.4	220.5	5.2	2407.0	403.7	41.4
42	M11-B4-42	111.6	220.0	5.2	2400.1	389.1	39.8
43	M11-B4-43	111.6	222.0	5.2	2415.3	396.5	40.5
• Table 10 represents the details of dimensions, mass, density, and the compressive failure strength for different cylindrical (ϕ×h=160×320mm) concrete samples of M-concrete.

**Table 10:** The details of dimensions, mass, density, and the compressive failure strength for different cylindrical (ϕ×h = 160 × 320 mm) concrete samples of M-concrete.

		Sample dimensions					

No.	Sample ID	Diameter, ϕ (mm)	Height, h (mm)	Mass of sample (kg)	Density, ρ (kg/m^3^)	Peak load, $F_{\max}$ (kN)	Compressive failure strength, $\sigma_{f}$ (MPa)
1	M16-B1-1	160.3	315.0	15.0	2362.7	834.2	41.3
2	M16-B1-2	160.0	315.3	15.1	2374.0	838.2	41.7
3	M16-B1-3	160.0	317.7	15.1	2367.0	824.7	41.0
4	M16-B1-4	160.0	315.7	15.1	2374.2	818.9	40.7
5	M16-B1-5	160.2	318.0	15.1	2362.0	809.5	40.2
6	M16-B1-6	160.0	315.7	15.1	2371.0	822.4	40.9
7	M16-B1-7	160.0	316.7	15.1	2376.1	843.5	42.0
8	M16-B1-8	160.0	316.3	15.1	2369.7	799.7	39.8
9	M16-B1-9	160.0	314.3	15.0	2372.1	805.0	40.0
10	M16-B1-10	160.0	315.0	15.0	2360.5	830.5	41.3
11	M16-B1-11	160.0	315.0	15.0	2370.9	831.1	41.3
12	M16-B1-12	160.0	318.0	15.2	2374.8	830.9	41.3
13	M16-B1-13	160.0	318.3	15.2	2367.3	848.3	42.2
14	M16-B2-14	160.0	310.0	14.7	2358.4	767.6	38.2
15	M16-B2-15	160.3	315.3	15.0	2357.3	740.6	36.7
16	M16-B2-16	159.8	316.3	15.1	2383.5	726.1	36.2
17	M16-B2-17	160.0	317.7	15.0	2354.5	738.4	36.7
18	M16-B2-18	160.2	316.7	15.1	2362.3	733.8	36.4
19	M16-B2-19	160.2	317.3	14.8	2312.5	757.3	37.6
20	M16-B2-20	160.3	314.0	14.9	2351.3	752.0	37.3
21	M16-B2-21	160.0	311.7	14.8	2363.1	749.7	37.3
22	M16-B2-22	160.8	318.0	15.1	2339.8	716.7	35.3
23	M16-B3-23	160.2	316.3	15.0	2357.5	763.9	37.9
24	M16-B3-24	160.0	316.7	15.2	2382.4	761.1	37.9
25	M16-B3-25	160.0	311.3	14.8	2371.0	749.9	37.3
26	M16-B3-26	160.3	315.0	15.0	2362.7	756.5	37.5
27	M16-B3-27	160.3	310.0	14.8	2370.4	801.1	39.7
28	M16-B3-28	160.3	317.3	15.1	2359.6	757.6	37.5
29	M16-B3-29	160.0	315.7	15.1	2372.6	781.2	38.9
30	M16-B3-30	160.0	316.7	15.1	2371.2	797.6	39.7
31	M16-B3-31	160.0	315.3	15.1	2377.2	786.0	39.1
32	M16-B3-32	160.0	313.3	15.0	2381.2	811.9	40.4
33	M16-B3-33	160.0	311.0	14.8	2363.7	809.9	40.3
34	M16-B4-34	160.0	317.2	15.2	2377.7	807.9	40.2
35	M16-B4-35	160.0	315.0	14.9	2359.7	806.1	40.1
36	M16-B4-36	160.0	314.7	15.0	2363.0	842.5	41.9
37	M16-B4-37	160.0	315.7	15.1	2372.6	772.3	38.4
38	M16-B4-38	160.0	315.3	15.1	2380.3	779.8	38.8
39	M16-B4-39	160.0	316.3	15.0	2361.8	786.1	39.1
40	M16-B4-40	160.2	313.3	14.9	2365.8	757.5	37.6
41	M16-B4-41	160.0	316.0	15.1	2376.6	781.0	38.8
• Table 11 represents the details of dimensions, mass, density, and the compressive failure strength for different cylindrical (ϕ×h=40×80mm) concrete samples of C-concrete.

**Table 11:** The details of dimensions, mass, density, and the compressive failure strength for different cylindrical (ϕ×h = 40 × 80 mm) concrete samples of C-concrete.

		Sample dimensions					

No.	Sample ID	Diameter, ϕ (mm)	Height, h (mm)	Mass of sample (g)	Density, ρ (kg/m^3^)	Peak load, $F_{\max}$ (kN)	Compressive failure strength, $\sigma_{f}$ (MPa)
1	C4-B1-1	39.0	81.0	232.0	2397.6	40.0	33.5
2	C4-B1-2	39.0	81.0	232.0	2397.6	46.4	38.9
3	C4-B1-3	39.0	81.0	231.0	2387.3	42.6	35.6
4	C4-B1-4	39.0	81.0	236.0	2439.0	41.9	35.1
5	C4-B1-5	39.0	81.0	232.0	2397.6	49.4	41.4
6	C4-B1-6	39.0	80.5	231.0	2402.1	45.8	38.3
7	C4-B1-7	39.0	81.0	232.0	2397.6	39.7	33.2
8	C4-B1-8	39.0	81.0	234.0	2418.3	55.1	46.1
9	C4-B1-9	39.0	81.0	234.0	2418.3	48.4	40.5
10	C4-B1-10	39.0	80.5	232.0	2412.5	41.2	34.5
11	C4-B1-11	39.0	81.0	233.0	2408.0	47.0	39.3
12	C4-B1-12	39.0	81.0	236.0	2439.0	57.4	48.0
13	C4-B1-13	39.0	80.5	233.0	2422.9	50.1	42.0
14	C4-B1-14	39.0	81.0	235.0	2428.6	47.9	40.1
15	C4-B1-15	39.0	80.5	232.0	2412.5	41.1	34.4
16	C4-B1-16	39.0	81.0	232.0	2397.6	56.2	47.0
17	C4-B1-17	39.0	81.0	237.0	2449.3	48.0	40.2
18	C4-B1-18	39.0	81.0	234.0	2418.3	48.2	40.3
19	C4-B1-19	39.0	81.0	234.0	2418.3	45.9	38.4
20	C4-B1-20	39.0	81.0	234.0	2418.3	41.8	35.0
21	C4-B1-21	39.0	81.0	233.0	2408.0	50.3	42.1
22	C4-B1-22	39.0	81.0	234.0	2418.3	41.5	34.7
23	C4-B1-23	39.0	82.0	237.0	2419.4	50.6	42.3
24	C4-B1-24	39.0	81.5	236.0	2424.0	47.6	39.8
25	C4-B1-25	39.0	81.5	240.0	2465.1	46.8	39.1
26	C4-B1-26	39.0	82.0	238.0	2429.7	50.3	42.1
27	C4-B1-27	39.0	82.0	238.0	2429.7	44.5	37.2
28	C4-B1-28	39.0	82.0	239.0	2439.9	44.9	37.6
29	C4-B1-29	39.0	81.5	239.0	2454.8	40.1	33.6
30	C4-B1-30	39.0	82.0	240.0	2450.1	52.4	43.9
31	C4-B1-31	39.0	82.0	236.0	2409.2	50.1	42.0
32	C4-B1-32	39.0	82.0	237.0	2419.4	47.0	39.3
33	C4-B1-33	39.0	81.5	236.0	2424.0	47.1	39.4
34	C4-B1-34	39.0	81.0	227.0	2346.0	54.5	45.6
35	C4-B1-35	39.0	81.0	228.0	2356.3	50.4	42.2
36	C4-B1-36	39.0	74.0	216.0	2443.4	42.2	35.3
37	C4-B1-37	39.0	80.5	232.0	2412.5	46.4	38.8
38	C4-B1-38	39.0	81.0	229.0	2366.6	59.0	49.4
39	C4-B1-39	39.0	81.0	233.0	2408.0	60.2	50.4
40	C4-B1-40	39.0	81.0	232.0	2397.6	49.3	41.3
41	C4-B1-41	39.0	81.0	235.0	2428.6	46.5	38.9
42	C4-B1-42	39.0	80.0	226.0	2364.8	41.1	34.4
43	C4-B1-43	39.0	81.0	237.0	2449.3	34.4	28.8
44	C4-B1-44	39.0	81.0	234.0	2418.3	37.2	31.2
• Table 12 represents the details of dimensions, mass, density, and the compressive failure strength for different cylindrical (ϕ×h=70×140mm) concrete samples of C-concrete.

**Table 12:** The details of dimensions, mass, density, and the compressive failure strength for different cylindrical (ϕ×h = 70 × 140 mm) concrete samples of C-concrete.

		Sample dimensions					

No.	Sample ID	Diameter, ϕ (mm)	Height, h (mm)	Mass of sample (g)	Density, ρ (kg/m^3^)	Peak load, $F_{\max}$ (kN)	Compressive failure strength, $\sigma_{f}$ (MPa)
1	C7-B1-1	69.5	140.0	1277.0	2404.4	135.6	35.7
2	C7-B1-2	69.5	140.0	1276.0	2402.5	136.7	36.0
3	C7-B1-3	69.5	140.0	1292.0	2432.6	123.0	32.4
4	C7-B1-4	69.5	140.0	1276.0	2402.5	172.5	45.5
5	C7-B1-5	69.5	139.5	1279.0	2416.8	187.1	49.3
6	C7-B1-6	69.5	140.0	1288.0	2425.1	175.4	46.2
7	C7-B1-7	69.5	140.0	1284.0	2417.6	183.1	48.3
8	C7-B1-8	69.5	140.0	1288.0	2425.1	145.6	38.4
9	C7-B1-9	69.5	140.0	1287.0	2423.2	140.8	37.1
10	C7-B1-10	69.5	139.0	1282.0	2431.2	125.1	33.0
11	C7-B1-11	69.5	140.0	1289.0	2427.0	131.3	34.6
12	C7-B1-12	69.5	139.0	1275.0	2417.9	135.1	35.6
13	C7-B1-13	69.5	140.0	1293.0	2434.5	135.4	35.7
14	C7-B1-14	69.5	140.0	1304.0	2455.2	140.3	37.0
15	C7-B1-15	69.5	140.0	1281.0	2411.9	116.0	30.6
16	C7-B1-16	69.5	139.5	1285.0	2428.1	157.1	41.4
17	C7-B1-17	69.5	140.0	1294.0	2436.4	122.2	32.2
18	C7-B1-18	69.5	139.5	1288.0	2433.8	151.4	39.9
19	C7-B1-19	69.5	139.5	1286.0	2430.0	151.1	39.8
20	C7-B1-20	69.5	140.0	1294.0	2436.4	162.5	42.8
21	C7-B1-21	69.5	140.0	1278.0	2406.3	170.8	45.0
22	C7-B1-22	69.5	140.0	1287.0	2423.2	115.0	30.3
23	C7-B1-23	69.5	140.0	1281.0	2411.9	128.3	33.8
24	C7-B1-24	69.5	141.0	1285.0	2402.3	129.5	34.1
25	C7-B1-25	69.5	140.0	1284.0	2417.6	188.9	49.8
26	C7-B1-26	69.5	139.5	1280.0	2418.7	172.6	45.5
27	C7-B1-27	69.5	140.0	1278.0	2406.3	157.3	41.5
28	C7-B1-28	69.5	140.0	1289.0	2427.0	187.0	49.3
29	C7-B1-29	69.5	139.5	1279.0	2416.8	174.7	46.1
30	C7-B1-30	69.5	140.0	1288.0	2425.1	179.7	47.4
31	C7-B1-31	69.5	140.0	1281.0	2411.9	169.9	44.8
32	C7-B1-32	69.5	140.2	1285.0	2416.0	117.1	30.9
33	C7-B1-33	69.5	140.0	1294.0	2436.4	184.4	48.6
34	C7-B1-34	69.5	140.0	1284.0	2417.6	184.5	48.6
35	C7-B1-35	69.5	140.0	1288.0	2425.1	165.8	43.7
36	C7-B1-36	69.5	140.0	1285.0	2419.4	152.8	40.3
37	C7-B1-37	69.5	140.0	1284.0	2417.6	169.1	44.6
38	C7-B1-38	69.5	140.0	1297.0	2442.0	110.8	29.2
39	C7-B1-39	69.5	140.0	1281.0	2411.9	118.7	31.3
40	C7-B1-40	69.5	135.0	1233.0	2407.5	165.1	43.5
41	C7-B1-41	69.5	134.0	1233.0	2425.5	145.5	38.3
42	C7-B1-42	69.5	140.0	1290.0	2428.9	154.3	40.7
• Table 13 represents the details of dimensions, mass, density, and the compressive failure strength for different cylindrical (ϕ×h=110×220mm) concrete samples of C-concrete.

**Table 13:** The details of dimensions, mass, density, and the compressive failure strength for different cylindrical (ϕ×h = 110 × 220 mm) concrete samples of C-concrete.

		Sample dimensions					

No.	Sample ID	Diameter, ϕ (mm)	Height, h (mm)	Mass of sample (kg)	Density, ρ (kg/m^3^)	Peak load, $F_{\max}$ (kN)	Compressive failure strength, $\sigma_{f}$ (MPa)
1	C11-B1-1	111.8	218.0	5.1	2385.4	348.6	35.5
2	C11-B1-2	111.9	220.0	5.2	2383.1	346.9	35.3
3	C11-B1-3	111.8	218.0	5.1	2388.2	350.0	35.7
4	C11-B1-4	111.9	217.0	5.1	2381.8	357.1	36.3
5	C11-B1-5	112.2	215.7	5.1	2370.7	324.5	32.8
6	C11-B1-6	111.9	219.0	5.1	2380.0	322.4	32.8
7	C11-B1-7	112.0	217.8	5.1	2385.2	319.6	32.4
8	C11-B1-8	111.9	220.0	5.2	2387.7	306.0	31.1
9	C11-B1-9	111.7	219.3	5.1	2395.5	308.5	31.5
10	C11-B1-10	111.8	212.0	5.0	2390.5	383.4	39.1
11	C11-B2-11	111.8	216.0	5.1	2419.8	365.2	37.2
12	C11-B2-12	111.7	220.0	5.2	2418.1	354.6	36.2
13	C11-B2-13	111.8	215.0	5.1	2416.3	356.6	36.3
14	C11-B2-14	111.7	216.0	5.1	2427.9	374.6	38.2
15	C11-B2-15	111.7	218.5	5.2	2416.5	358.5	36.6
16	C11-B2-16	111.8	217.0	5.2	2422.7	365.7	37.3
17	C11-B2-17	112.0	217.7	5.2	2417.0	344.1	34.9
18	C11-B3-18	111.8	222.0	5.2	2389.7	348.1	35.5
19	C11-B3-19	111.8	219.7	5.1	2369.3	352.4	35.9
20	C11-B3-20	112.0	222.2	5.2	2384.5	367.5	37.3
21	C11-B3-21	112.2	216.5	5.1	2395.6	376.8	38.1
22	C11-B3-22	111.8	219.0	5.1	2381.5	339.0	34.5
23	C11-B3-23	112.0	221.0	5.2	2386.0	302.5	30.7
24	C11-B3-24	111.7	221.0	5.2	2396.5	333.4	34.0
25	C11-B3-25	111.8	220.0	5.2	2384.6	449.3	45.8
26	C11-B4-26	111.9	221.0	5.2	2375.1	394.6	40.1
27	C11-B4-27	111.8	221.0	5.2	2380.7	390.2	39.7
28	C11-B4-28	111.5	215.7	5.1	2411.0	388.8	39.8
29	C11-B4-29	111.8	219.0	5.2	2422.9	379.3	38.6
30	C11-B4-30	112.0	219.0	5.2	2409.6	392.5	39.8
31	C11-B4-31	112.2	218.0	5.1	2379.1	365.3	36.9
32	C11-B4-32	112.0	223.0	5.2	2377.3	402.1	40.8
33	C11-B4-33	112.0	214.0	5.1	2409.0	375.9	38.2
34	C11-B4-34	111.5	221.7	5.2	2400.3	369.5	37.8
35	C11-B4-35	111.9	219.0	5.2	2396.8	401.4	40.8
36	C11-B4-36	111.8	221.3	5.2	2411.1	373.4	38.0
37	C11-B4-37	112.0	223.7	5.2	2363.1	374.9	38.1
38	C11-B4-38	112.0	219.0	5.1	2374.9	403.5	41.0
39	C11-B4-39	111.8	220.0	5.2	2399.9	392.4	40.0
40	C11-B4-40	112.0	221.8	5.2	2377.4	395.0	40.1
41	C11-B4-41	112.3	222.0	5.2	2363.9	383.0	38.7
42	C11-B4-42	112.1	218.7	5.1	2372.0	401.9	40.7
43	C11-B4-43	112.0	220.7	5.2	2406.2	384.1	39.0
44	C11-B4-44	111.8	222.7	5.3	2411.9	411.9	42.0
• Table 14 represents the details of dimensions, mass, density, and the compressive failure strength for different cylindrical (ϕ×h=160×320mm) concrete samples of C-concrete.

**Table 14:** The details of dimensions, mass, density, and the compressive failure strength for different cylindrical (ϕ×h = 160 × 320 mm) concrete samples of C-concrete.

		Sample dimensions					

No.	Sample ID	Diameter, ϕ (mm)	Height, h (mm)	Mass of sample (kg)	Density, ρ (kg/m^3^)	Peak load, $F_{\max}$ (kN)	Compressive failure strength, $\sigma_{f}$ (MPa)
1	C16-B1-1	160.0	313.5	14.9	2362.3	683.2	34.0
2	C16-B1-2	160.0	316.0	15.0	2357.1	711.5	35.4
3	C16-B1-3	160.0	316.0	15.1	2375.7	686.8	34.2
4	C16-B1-4	160.0	312.3	14.8	2362.9	786.6	39.1
5	C16-B1-5	160.0	313.8	14.9	2364.8	743.6	37.0
6	C16-B1-6	160.0	314.0	15.0	2381.1	752.3	37.4
7	C16-B1-7	160.0	312.3	14.9	2368.3	756.8	37.6
8	C16-B1-8	160.0	315.0	15.0	2369.6	767.5	38.2
9	C16-B1-9	160.0	311.7	14.8	2365.8	754.2	37.5
10	C16-B1-10	160.0	318.0	15.1	2357.8	701.1	34.9
11	C16-B2-11	160.0	314.3	15.0	2368.4	735.3	36.6
12	C16-B2-12	160.0	316.2	15.2	2395.3	767.9	38.2
13	C16-B2-13	160.0	315.2	15.2	2402.5	739.0	36.8
14	C16-B2-14	160.0	318.3	14.9	2334.6	743.6	37.0
15	C16-B2-15	160.0	311.0	15.1	2410.8	762.1	37.9
16	C16-B2-16	160.0	316.0	15.3	2413.8	748.5	37.2
17	C16-B2-17	160.0	317.5	15.2	2383.9	697.3	34.7
18	C16-B2-18	160.0	315.2	15.2	2405.7	766.9	38.1
19	C16-B2-19	160.0	316.3	15.1	2381.1	753.9	37.5
20	C16-B2-20	160.0	312.0	15.0	2394.2	708.5	35.2
21	C16-B3-21	160.0	314.0	15.1	2387.2	766.7	38.1
22	C16-B3-22	160.0	311.2	14.9	2375.7	742.5	36.9
23	C16-B3-23	160.0	308.0	14.8	2383.1	738.7	36.7
24	C16-B3-24	160.0	315.0	15.2	2399.2	746.5	37.1
25	C16-B3-25	160.0	315.8	15.1	2375.3	720.5	35.8
26	C16-B3-26	160.0	313.0	15.0	2380.7	750.5	37.3
27	C16-B3-27	160.0	310.0	14.9	2387.3	696.0	34.6
28	C16-B3-28	160.0	312.0	14.8	2366.6	729.8	36.3
29	C16-B3-29	160.0	311.0	14.9	2378.8	733.0	36.5
30	C16-B3-30	160.0	313.2	15.0	2381.2	718.9	35.8
31	C16-B4-31	160.0	316.0	15.2	2385.9	739.3	36.8
32	C16-B4-32	160.0	317.3	15.3	2391.3	741.9	36.9
33	C16-B4-33	160.0	313.0	15.1	2397.5	719.8	35.8
34	C16-B4-34	160.0	315.0	15.3	2413.8	745.3	37.1
35	C16-B4-35	160.0	316.3	15.2	2395.0	765.2	38.1
36	C16-B4-36	160.0	313.2	15.1	2404.1	789.4	39.3
37	C16-B4-37	160.0	314.0	15.2	2406.3	762.7	37.9
38	C16-B4-38	160.0	315.0	15.1	2389.9	774.6	38.5
39	C16-B4-39	160.0	314.2	15.3	2417.9	716.9	35.7
40	C16-B4-40	160.0	316.8	15.2	2392.8	665.4	33.1

## 2 Experimental Design, Materials and Methods

### 2.1 Experimental design and methods

To obtain the dataset presented in this paper, several types of experiments were performed.

The first set of experiments consisted in non-destructive tests (i.e. Acoustic Emission (AE) measurements) performed to determine the velocity of P-waves through hardened concrete samples. This velocity was then used to calculate the dynamic elastic modulus of concrete samples. These P-wave tests were performed by using a MISTRAS-2001 AE system manufactured by Physical Acoustics Corporation (PAC) to record AE signals artificially generated from the breaking of a Pencil lead (Hsu-Nielsen source) at the ends of concrete cylindrical samples. Full details on the measurement procedure can be found in ref. [1], and the data are reported on Table 1. In this table, the values of apparent densities and moisture contents of all sample sizes and all mixtures are also presented. The apparent density of each sample was calculated by dividing the mass of the concrete sample (m) by its volume $\left(V=h\frac{\pi \phi^{2}}{4}\right)$, conforming to the French standard NF EN 12390-7 [3]. All the weight (m), the diameter (ϕ), and the height (h) of the concrete samples were measured before each loading test, for a total of 527 densities values determined. After the compression tests, 10 deformed concrete specimens for each sample size and each concrete group were collected and weighted for determining the moisture content. A series of 120 moisture content measurements were performed following the procedure described in the regulation NF EN 12390-7 [3] and [4]. In the present study, the moisture content of the hardened concrete samples was calculated as the ratio of the mass difference before and after drying, divided by the dried mass (i.e. the mass ratio of water to solid phases in the sample). All the details about the density and moisture content measurements have been given in ref. [2].

The second type of experimental data was obtained from image analysis of internal sections. This work aimed at investigating the microstructural characteristics of hardened concrete samples: the microstructural characteristics length scales (i.e. $\xi_{g}$- the global autocorrelation length describing the internal microstructure as a whole, and $\xi_{p}$- the autocorrelation length of the pore structure); the corresponding integral ranges $X_{0,g}$ and $X_{0,p}$; the mean pore diameter $\langle d_{p}\rangle$; the maximum pore diameter $\left(d_{p,\text{max}}\right)$; and the porosity $\left(p_{0}\right)$. For this purpose, one sample for each size and each concrete group, for a total of 12 concrete samples, were used. These intact (unloaded) samples were firstly cut into four pieces. Next, the surfaces of these four pieces were polished and then scanned by a flatbed scanner with a resolution of 1200dpi corresponding to a 21.17μm pixel size. After that, these scanned images were analyzed to characterize the internal microstructures of the concrete samples. To examine the pore structure of the hardened concrete samples, sectional surfaces were treated to enhance the contrast between pores and solid components (aggregates, cement paste) by filling depressions with a calcium carbonate paste. Thanks to the white color of this paste, the pores were easily distinguished from the aggregates and the hardened cement paste after applying a manual contrast enhancement and thresholding. After that, the two-dimensional (2D) pore size distributions obtained from the binary images of pores. They were converted to three-dimensional (3D) pore size distributions by using the stereological 2D → 3D conversion method of Saltykov [5,6]. Full details on these image analysis procedures and the definition of the measured variables can be found in refs. [1,2].

Finally, uniaxial compression tests were performed on concrete cylinders to analyze the mechanical behavior under compressive loading and to collect the values of compressive failure strength for different samples. All these loading tests were performed under a load control protocol in conformity with the regulation NF EN 12390-3 [7]. In this work, two compression machines (of different stiffness and loading capacity) (see Fig. 2 c and d in ref. [2]) were used due to the very different concrete sample sizes. These two machines, calibrated and certified by the French Accreditation Committee (COFRAC), are stiff enough to conduct reliable strength experiments on our concrete samples. A loading rate of 0.5 MPa/s was applied for all compression tests and kept constant during the test. The loading and data recording were automatically stopped when the load fell below 50% of the peak load. During the test, both the load(F) and the axial displacement (δ) of the bottom steel platen of the compression machine were recorded. The displacement δ was measured by one Linear Variable Differential Transducer (LVDT) attached on the frame of the machine. Among the 527 compression tests performed, a technical problem did not allow to record the LVDT data for 35 tests on 160mm-samples of C-concrete mixture. The stress-strain records are therefore not provided for these 35 tests. Note that both the concrete sample and the compression machine were deforming during loading. As a result, the axial displacement (δ) recorded directly from the test was considered as a sum of the genuine axial shortening of the sample $\left(\Delta_{sp}\right)$ and the elastic deformation of the loading frame $\left(\Delta_{fr}\right)$. By performing calibration tests on reference samples of known elastic moduli (i.e. an Aluminum sample for the machine shown in Fig. 2c in ref. [2], and a stainless steel sample for the machine shown in Fig. 2d in ref. [2]), the displacement $\Delta_{fr}$ of each machine was determined as a function of the applied load. This displacement was then eliminated from the measured displacement (δ) to obtain $\Delta_{sp}$. The axial strain (ε) of the concrete sample was calculated by dividing the displacement $\Delta_{sp}$ (after calibration) by the height of the sample (h).

Additional experimental details can be found in Ref. [1].

### 2.2 Materials and concrete specimens

For our experimental investigations, 539 concrete specimens of 3 different concrete mixtures and 4 different sizes were fabricated. Twelve (12) of these concrete samples were selected for the microstructural characterization from image analysis (Table 2) and the 527 remaining samples were used for the uniaxial compression tests. All concrete samples were produced using CEM I 52.5N ordinary Portland cement (satisfying the standard NF EN 197-1 [8]), a variable amount of fine (i.e. natural sand) and of coarse (i.e. natural gravel) aggregates, and tap water. In this work, the weight method in accordance with the French standard NF EN 206-1 [9] was applied for preparing the three concrete mixtures based on three different aggregate sizes (see Fig. 2b in ref. [2]). The corresponding abbreviations for identifying each concrete group indicate the size of aggregates used for concrete fabrication: Fine aggregate (F-mixture), Medium aggregate (M-mixture), and Coarse aggregate (C-mixture). The size distribution of these aggregates, investigated from a sieving analysis following the regulation NF EN 933-1 [10], are displayed in Fig. 1 inf ref. [2], while the mix proportions of the three concrete mixtures are reported in Table 1 in ref. [2]. The volume fraction of aggregates was calculated by subtracting the sum of the volume fraction of cement and water from the 1m^3^ of concrete. For this calculation, a cement density of 3100 kg/m^3^ following the product catalog and a water density of 1000 kg/m^3^ were used. The volume fraction of aggregates was approximately 0.7 m^3^/m^3^ for the coarser mixes (M- and C- concretes) and 0.63 m^3^/m^3^ for the F-concrete. All the concrete samples were cylinders with a constant height-to-diameter ratio, h/ϕ=2. For a specified concrete mixture, the diameter (ϕ) ranged as follows: 40, 70, 110 and 160 mm (see Fig. 2a in ref. [2]). The details of casting and curing procedures for all our concrete samples have been described in [1].

### 2.3 Sample labeling

As mentioned above, the dataset herein consists of the text *(.txt)* files of stress-strain data provided as supplementary files. Each text file provides uniaxial compressive stress and strain values recorded during the loading test of each concrete sample. The names of this text file and of the “Sample ID” in the 12 tables (from Table 3 to Table 14) are the same. The name format of the *txt* files and for the identification of the concrete samples (i.e. Sample ID) is **XY-Z-K**. “X” indicates the concrete mixture and can be either F, M, or C (see above). “Y” is the diameter of the concrete cylindrical sample (in cm) and can be either 4, 7, 11 or 16. “Z” is related to the batch number. For each concrete mixture, all the 40 × 80- and 70 × 140 mm samples were produced from a single batch. Hence, “Z” is always B1 for these sample sizes. However, for the 110 × 220- and 160 × 320 mm samples it can be either B1, B2, B3, or B4. Indeed, as the result of a limited capacity of the concrete mixer, it was not possible to prepare all the large samples (110 mm and 160 mm in diameter) from a single batch. Instead, four batches were required to prepare all the 110 mm samples, as well as for the 160 mm samples. These different batches can be considered a priori as a source of strength variability for each concrete group, and this has been taken into account in our analysis in Ref. [1]. The last term “K” is the sample number. For instance, the name of txt file or Sample ID “F4-B1-30” is related to a F-concrete sample with a diameter of 4 cm fabricated from the corresponding batch B1 and its sample number, for this size and concrete mix, is 30.

## Declaration of Competing Interest

The authors declare that they have no known competing financial interests or personal relationships which have, or could be perceived to have, influenced the work reported in this article.

## Appendix Supplementary materials

Image, application 1
